# The dual role of the gut microbiota in breast cancer: from pathogenic mechanisms to emerging therapeutic

**DOI:** 10.3389/fmicb.2025.1712240

**Published:** 2025-12-01

**Authors:** Yihong Zhao, Ye Zhao, Jingyi Shi, Bingrui Guan, Meihan Long, Zhenzhen Sun, Shanlin Jin, Yan Ye, Tiebao Chang, Yunhe Fu, Huihua Zheng, Guanghong Xie

**Affiliations:** 1 Department of Dermatological, China-Japan Union Hospital of Jilin University, Changchun, Jilin, China; 2 Department of Clinical Veterinary Medicine, College of Veterinary Medicine, Jilin University, Changchun, Jilin, China; 3 Department of Veterinary Medicine, College of Veterinary Medicine, Zhejiang Agricultural and Forestry University, Hangzhou, Zhejiang, China

**Keywords:** breast cancer, gut microbiota, microbiota metabolism, development, treatment

## Abstract

Recent studies indicate that breast cancer (BC) has surpassed lung cancer as the most frequently diagnosed cancer worldwide and remains the leading cause of cancer-related death among women. Although advances in medical therapy have improved survival rates for BC patients, its etiology is highly complex and necessitates further investigation into the underlying biological mechanisms and risk factors. The gut microbiota, a critical microbial ecosystem within the human body, is increasingly recognized as being closely linked to the initiation and progression of various diseases, including BC. It plays a pivotal role in key physiological processes such as estrogen metabolism and immune regulation. Gut microbiota dysbiosis may contribute to pathological alterations that influence BC development, progression, and response to treatment. This review summarizes the current understanding of the gut microbiota’s role in BC pathogenesis and therapeutic outcomes, highlighting recent advances. We discuss the mechanistic pathways by which the gut microbiota affects BC, including modulation of estrogen metabolism, immune system regulation, and impacts on treatment efficacy, thereby providing a theoretical framework for further exploration of disease mechanisms and therapeutic interventions. Moreover, by synthesizing and critically analyzing existing evidence, this review identifies emerging research directions and potential therapeutic targets, offering valuable insights for future translational research and clinical applications.

## 1 Introduction

Breast cancer (BC) is one of the most prevalent malignant tumors in women and has become the most frequently diagnosed cancer globally (Bagher et al., 2018). In recent years, there has been a growing trend toward earlier age at diagnosis (Kaszak et al., 2018). The causes of BC are complex and varied, making it challenging to pinpoint when it begins. Factors such as ultraviolet light exposure, genetic predispositions, estrogen production, family history, and obesity can all contribute to the development of BC. Based on molecular characteristics, BC is generally categorized into luminal A, luminal B, HER2 overexpression, and triple-negative breast cancer (TNBC) (Edward et al., 2022; Liedtke et al., 2008). Additionally, from a histopathological perspective, it can be classified as invasive or non-invasive BC. Furthermore, BC can also be staged as 0, I-III, or IV based on its clinical behavior (Trayes and Cokenakes, 2021). Recent years, programmed death ligand 1 (PD-L1) inhibitors are effective in tumor response and PD-L1 also serves as an immune checkpoint for cancer (Li et al., 2021; Mo et al., 2024). Cancer patients with higher gut microbiota diversity exhibit improved responses to immune checkpoint inhibitor (ICI) therapy. The gut microbiota can directly downregulate the expression of immune checkpoints on immune cells and mobilize anti-tumor immune cells through the production of metabolites such as indole, inosine, and short-chain fatty acids (SCFAs) (Guillot et al., 2023).

Humans and other mammals harbor complex gastrointestinal microbiota, commonly referred to as the gut microbiota (Caballero-Flores et al., 2023). The gut microbiota consists of a diverse array of interacting bacteria, archaea, bacteriophages, eukaryotic viruses, and fungi that coexist within the host’s gastrointestinal tract and is shaped by the host’s lifestyle and genetic makeup (Caballero-Flores et al., 2023; Rooks and Garrett, 2016). Recent research has indicated that the gut microbiota plays a role in modulating various inflammatory diseases, metabolic disorders, and cancers. By analyzing the gut microbiota composition in clinical cohorts of lung cancer patients, studies have revealed distinct microbial signatures associated with lung cancer, suggesting that gut microbiota characteristics may serve as predictors for early detection (Zheng et al., 2020). In addition to the intrinsic functional capabilities of the gut microbiota, their metabolic activities also contribute to host physiological and pathological processes (Wang et al., 2023). In host metabolism, the gut microbiota plays a crucial role in breaking down dietary components such as resistant starch, pectin, oligosaccharides, and other non-digestible carbohydrates into biologically active compounds, which are subsequently fermented into SCFAs, including acetic, propionic, and butyric acids (Gomaa, 2020; Sanna et al., 2019). In a mouse model of prostate cancer fed a high-fat diet, alterations in the gut microbiota were observed. Antibiotic treatment reduced the production of SCFAs by the gut microbiota, whereas exogenous administration of SCFAs was found to promote prostate cancer progression through elevation of insulin-like growth factor 1 (IGF-1) levels (Matsushita et al., 2021). Besides, bile acid, a byproduct of microbiota, causes an imbalance in gut microbiota and contributes to the development of intestinal cancer (Cao et al., 2017).

In recent years, targeted modulation of the gut microbiota has demonstrated promising results in enhancing the efficacy of cancer immunotherapy. Studies have shown that gut microbiota dysbiosis and alterations in microbial metabolite profiles lead to increased levels of lipopolysaccharide (LPS), which translocates into the bloodstream through a compromised intestinal barrier and triggers mastitis. Supplementation with depleted probiotics or beneficial metabolites, such as *Lactobacillus reuteri*, secondary bile acids, and hexadecanamide, has been shown to alleviate mastitis in mouse models. These findings confirm the existence of the “gut–mammary” axis linking the gut and the mammary gland (Zhao et al., 2022; Zhao et al., 2023; He et al., 2024; Zhao et al., 2023; Bao et al., 2023). Similarly, the gut microbiota influences the development process of BC (Cao et al., 2024). Compared with postmenopausal healthy individuals, the relative abundance of 45 bacterial species was significantly altered in postmenopausal BC patients. Among these, 38 species such as *Escherichia coli* and *Enterococcus gallinarum*—were enriched, whereas the abundance of 7 species such as *Eubacterium eligens* and *Lactobacillus vaginalis* was reduced (Zhu et al., 2018). The reduction of *Akkermansia muciniphila* and butyric acid levels in the gut can accelerate the growth of BC (Cui et al., 2025).

Given these findings, the association between gut microbiota and BC has increasingly attracted research attention. This review outlines the mechanisms by which the gut microbiota contributes to BC progression and discusses its potential applications in cancer treatment, highlighting the significance of gut microbiota in BC and providing insights for clinical therapeutic strategies.

## 2 Gut microbiota influences BC development and underlining mechanisms

Multiple studies in clinical cohorts have demonstrated that cancer patients exhibit structural imbalances in their gut microbiota (Kartal et al., 2022; Hou et al., 2021). By collecting fecal samples from patients with clinical BC and performing microbiota sequencing analysis, studies have demonstrated that the gut microbiota can influence BC progression (Zhu et al., 2018). An analysis was conducted on 267 individuals, including premenopausal breast cancer (Pre-BC) patients, postmenopausal breast cancer (Post-BC) patients, and age-matched female controls. The diversity, composition, and metabolic functional pathways of the gut microbiota were assessed. Results showed that α-diversity was significantly reduced in Pre-BC patients, and significant differences in both microbial composition and functional pathways were observed between breast cancer patients and control females (Hou et al., 2021). Therefore, targeted detection of the gut microbiota may be used for the diagnosis of BC.

### 2.1 The regulatory effect of gut microbiota on estrogen metabolism

**Figure 1:**
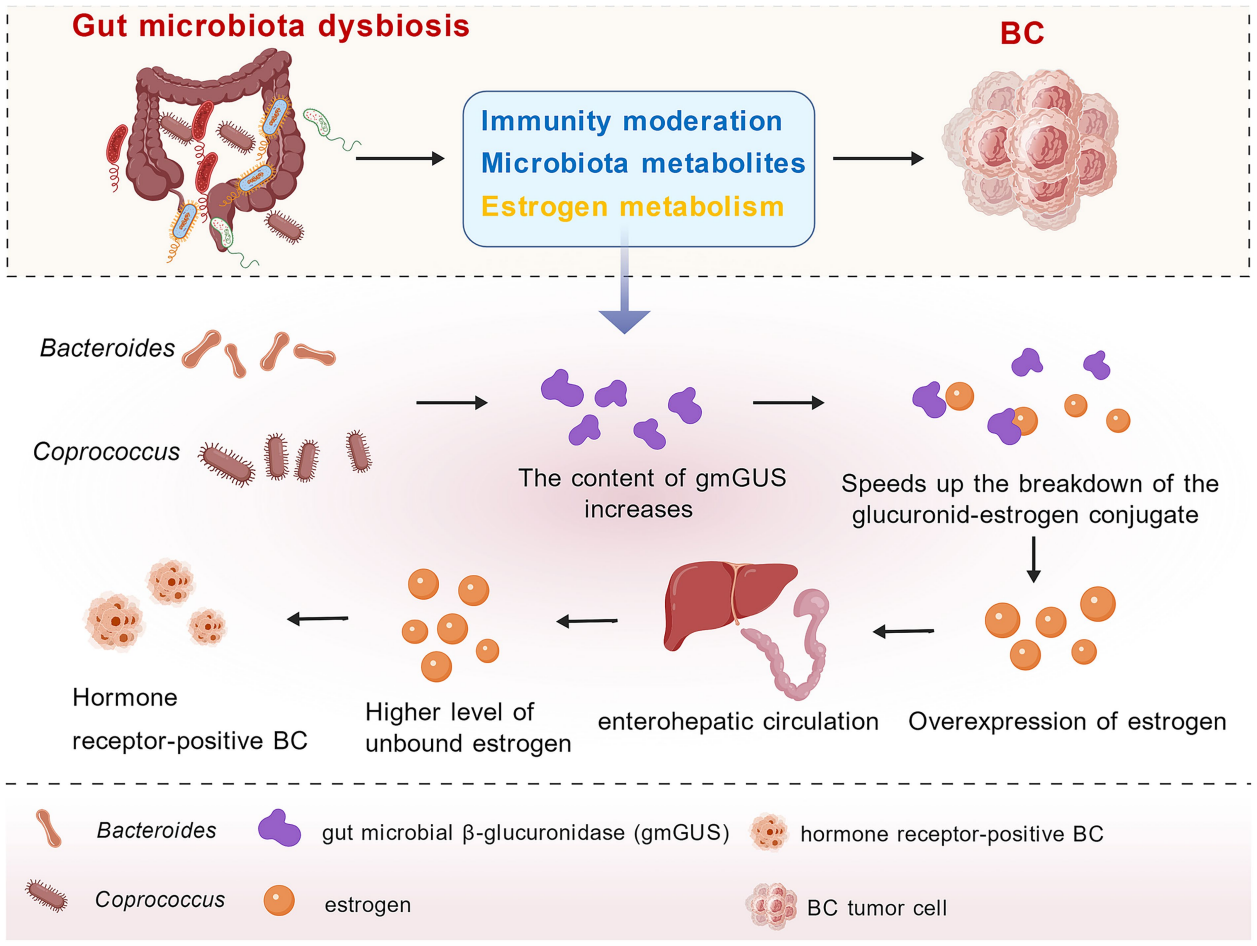
The regulatory effect of gut microbiota on estrogen metabolism. *Bacteroides* and *Coprococcus* can produce gmGUS to regulate estrogen metabolism. When the gut microbiota is imbalanced, excessive gmGUS causes free estrogens to enter the bloodstream via the enterohepatic circulation, leading to elevated estrogen levels in the host and increasing the risk of hormone receptor-positive BC (all figures are created with BioGDP.com).

Mammary glands are composed of acini, ducts, and stroma, with healthy mammary epithelial cells arranged in a highly organized architecture. The coordinated processes of cell proliferation and apoptosis jointly maintain a dynamic equilibrium within the tissue (McGhee and Steele, 2020). Throughout the various phases of the menstrual cycle, hormonal fluctuations regulated by estrogen and progesterone exert significant influence on the physiological state of the mammary gland (McGhee and Steele, 2020; Alex et al., 2020). Reducing estrogen stimulation may help reduce the risk of cancer (Perez Alenza et al., 2000; Burrai et al., 2015). Chronic psychological stress induces elevated levels of bile acids, which are accompanied by increased estradiol concentrations. The administration of Si-Ni-San for the treatment of psychological disorders can suppress estradiol levels through modulation of the FXR/EST signaling pathway, thereby inhibiting BC progression (Zhang et al., 2023). A recent study has demonstrated that the gastrointestinal microbiota influences estrogen metabolism and modulates the balance of hormone circulation and secretion, thereby affecting BC development (Laborda-Illanes et al., 2020; Yang et al., 2017). In the host gut, some bacteria produce β-glucuronidase, including *Bacteroides* and *Coprococcus* (Hu et al., 2023). Gut microbial β-glucuronidase (gmGUS) is a key factor in regulating host estrogen metabolism. GmGUS activates estrogen and increases the availability of intestinal estrogen for absorption in the blood (Hu et al., 2023; Fuhrman et al., 2014). Under normal health conditions, the interactions between gmGUS and estrogen help to keep physiological estrogen levels stable in the body. However, when the gut microbiota is disrupted, this balance is thrown off. A decrease in microbiota abundance causes an excessive rise in gmGUS, which speeds up the breakdown of the glucuronid-estrogen conjugate. This process leads to an overproduction of estrogen and its reversion to its free form (Baker et al., 2017; Whitaker, 1960; Rosenberg et al., 2016). Estrogen is later reabsorbed into the bloodstream via enterohepatic circulation, leading to a higher level of unbound estrogen in the body, which ultimately raises the occurrence of hormone receptor-positive BC (Yang et al., 2017; Hu et al., 2023; Whitaker, 1960) (Figure 1). Although targeting gut microbiota dysbiosis to restore estrogen homeostasis holds promise as a novel therapeutic strategy, this approach primarily centers on the “gut microbiota–gmGUS–estrogen” axis, reflecting a relatively narrow scope. It fails to adequately account for other well-established risk factors—such as genetic mutations and reproductive history—and does not comprehensively address the mechanisms by which the gut microbiota influences breast cancer through non-estrogen-mediated pathways, including immune regulation. Additional evidence will be necessary to validate the feasibility and broader applicability of this strategy.

### 2.2 The influence of immune regulation mediated by gut microbiota on BC

In the advanced stages of BC, the primary treatment modalities are chemotherapy and radiation therapy, both of which can exert significant systemic side effects. In contrast, immunotherapy targets the host immune system to promote the elimination of tumor cells through activation of immune responses. This therapeutic approach encompasses immune checkpoint inhibitors (ICIs), adoptive immune cell therapy, and tumor vaccines.

The suppression and reprogramming of the immune system are critical factors in tumor initiation and progression. Immunotherapy aims to reactivate anti-tumor immune cells and counteract the immune evasion mechanisms employed by tumors. Recently, researchers have investigated the influence of gut microbiota on cancer immunotherapy, as it can modulate anti-tumor immune responses and affect treatment outcomes through multiple pathways. In clinical trials evaluating the efficacy of PD-1 antibodies in metastatic melanoma, non-small cell lung cancer, and renal cell carcinoma, patients who responded favorably to PD-1 blockade were found to exhibit higher gut microbial diversity (Gharaibeh and Jobin, 2019). *Lactobacillus joseri* and *Clostridium sporogenes* cooperate to produce indolepropionic acid (IPA), which improves the response of various cancers, including melanoma, BC and colorectal cancer, to immune checkpoint therapy by promoting the production of CD8^+^ T cell progenitor cells (Tpex) (Jia et al., 2024). Compared with healthy dogs, the abundance of *Bacteroides*, *Helicobacter* and *Cetobacterium* in the intestinal microbiota of dogs with BC is significantly increased (Zheng et al., 2022). *Helicobacter* has been found to have both beneficial and harmful effects on cancer development in mice, which vary according to bacterial species, host immune status, and cancer type (Hand and Overacre-Delgoffe, 2022). The trimethylamine N-oxide (TMAO) produced by gut microbiota metabolism can activate CD8^+^ T cell-mediated anti-tumor immunity and enhance the efficacy of immunotherapy in mouse models of triple-negative BC (Wang et al., 2022). The immune responses driven by the gut microbiota enhance anti-tumor immunity (Lam et al., 2021). However, most of these studies are based on correlational observations, and it remains unclear whether specific microbiota act as the “cause” or the “effect” of treatment outcomes. Meanwhile, the underlying mechanisms linking the microbiota and host responses are highly complex and cannot be oversimplified. Future research should integrate prospective clinical cohorts with multi-omics technologies to elucidate the causal roles of key microbial taxa and their metabolites in host physiology and therapeutic responses (Table 1).

**Table 1:** Summary of clinical trials related to gut microbiota and cancer.

Disease	Study model	Study design	The role of gut microbiota
Lung cancer	Humans: China	16S rRNA amplicon sequencing and lung cancer	It revealed the microbiota profile of lung cancer patients (Zheng et al., 2020).
Breast cancer	Humans: China	Metagenomic DNA sequencing and breast cancer	The relative abundance of 45 bacterial species in postmenopausal BC patients changed (Zhu et al., 2018).
Pancreatic cancer	Humans: Spain	16S rRNA amplicon sequencing and pancreatic cancer	Cancer patients exhibit different structures of gut microbiota (Kartal et al., 2022).
Breast cancer	Humans: China	16S rRNA amplicon sequencing and breast cancer	α-diversity was significantly reduced in Pre-BC patients (Hou et al., 2021).
Breast cancer	Humans: China	Metagenomic DNA sequencing and breast cancer	Higher gut microbiota diversity is associated with improved outcomes in neoadjuvant chemotherapy (Li et al., 2022).
Breast cancer	Humans: Denmark	Cox proportional hazards regression models and breast cancer	Obesity is an independent prognostic factor for developing distant metastases and for death as a result of breast cancer (Ewertz et al., 2011).
Breast cancer	Humans: France and Italy	Metagenomic DNA sequencing and breast cancer (NCT01993498)	Specific gut commensals that are overabundant in BC patients compared with healthy individuals negatively impact BC prognosis (Terrisse et al., 2021).
Anti-PD-(L)1 monotherapy for unresectable or metastatic solid tumors	Humans: Korea	An anti-PD-1 inhibitor with FMT from anti-PD-1 responderin13 patientswithanti-PD-1-refractory advanced solid cancers (NCT04264975)	FMTwithanti-PD-1showsbenefitsinadvancedsolidcancers resistant to anti-PD-1 (Kim et al., 2024).
Melanoma	Humans: Canada	The safety and response to anti-PD1 combined with FMT using healthy donor stool as first-line treatment (MIMic, NCT03772899)	Oral FMT capsules in this small cohort was safe and appeared to improve clinical outcomes and possibly avoid primary resistance (Hadi et al., 2025).

### 2.3 The association between metabolites related to the gut microbiota and the risk of BC

Increased permeability of the gut and mammary barriers raises the possibility of gut microbiota translocation into the mammary gland, which in turn alters the microbial composition within this tissue (Chen et al., 2020). The gut microbiota primarily colonizes the mucosal surfaces of the colon and ileum. When the intestinal mucosal barrier is compromised—such as during inflammation, cancer, or microbial dysbiosis—certain microorganisms, particularly opportunistic pathogens like *Enterococcus* and *E. coli*, as well as limited quantities of beneficial bacteria, can translocate across the intestinal barrier, gain access to the lamina propria or interact directly with intestinal epithelial cells, and subsequently disseminate to distant organs via the bloodstream, including the mammary gland. FMT experiments from dairy cows to mice demonstrated that the microbial composition in the mammary glands of mice receiving microbiota from mastitis-affected cows (M-FMT) significantly differed from that of mice receiving microbiota from healthy cows (H-FMT), with increased abundances of *Escherichia_Shigella* and *Bacteroides*. On day 25 post-FMT, green fluorescent protein-labeled *E. coli* (GFP-*E. coli*) was administered orally. Strong GFP signals were detected in the mammary glands of mice in the M-FMT group, whereas only low levels of GFP-*E. coli* signals were observed in the control group and the H-FMT group (Zhao et al., 2022). Enteric *Stenotrophomonas maltophilia* can translocate from the intestine to the mammary gland via the gut-mammary axis and activate the calcium–ROS–AMPK–mTOR–autophagy signaling pathway, thereby inducing mastitis.

In addition, the various metabolites produced by the gut microbiota are closely related to the risk of BC. Besides the β-glucuronidase mentioned earlier, which is related to estrogen metabolism, one of the most well-known metabolites – SCFAs, which play an important role in cancer and metastasis. Butyrate increases the histone lysine acetylation effect (H3K27ac) in the promoter region and human CD8^+^ T cells, thereby promoting the expression of PD-1/CD28 and enhancing the efficacy of anti-PD-1 therapy (Zhu et al., 2023). At the same time, butyrate supplementation promotes the expression of anti-tumor cytokines in cytotoxic CD8^+^ T cells by regulating the T cell receptor (TCR) signaling pathway, enhancing anti-tumor immunity (Zhu et al., 2023). The intestinal symbiotic bacterium *Roseburia* produces butyric acid, which inhibits the translocation of opportunistic pathogenic bacteria to the mammary gland by restoring intestinal barrier integrity (Zhao et al., 2022). In addition, the gut microbiota also produces other metabolites with potential carcinogenic or tumor-suppressing effects. Indole and its derivatives can regulate the immune system function and indirectly affect the occurrence and development of BC. In the early stage of BC, the indole biosynthesis of the intestinal microbiome is inhibited. Supplementing IPA can inhibit the proliferation of 4 T1 cells, increase cellular oxidative stress and cellular energy stress, and reduce the number of cancer stem cells, but it has no cytotoxicity to normal human primary fibroblasts (Sári et al., 2020). *L. johnsonii* and *C. sporogenes* collaborate to produce IPA, which modulates CD8^+^T cell stemness by increasing H3K27 acetylation at the Tcf7 super-enhancer, thereby playing the role in anti-tumor (Jia et al., 2024). In summary, there exists a gut-mammary axis in the host, indicating that the gut microbiota can translocate to the mammary gland and influence the breast microenvironment and BC development through their metabolites—such as butyrate and indole derivatives—by modulating intestinal barrier function and immune responses. However, most current evidence is derived from animal models, and the universality and relevance of this axis in humans remain unclear. The specific pathways, frequency of microbial translocation, and causal role of the gut microbiota in human BC development require direct experimental validation.

## 3 The role of microbiota in tumor treatment

When treated with ICI, specific gut microbiota can enhance systemic immunity and promote intratumoral immune cell infiltration. Approaches such as FMT, probiotic supplementation, and dietary interventions may be used to modulate the gut microbiota for cancer therapy (Helmink et al., 2019).

### 3.1 The influence of gut microbiota on the efficacy and side effects of chemotherapy

Chemotherapy is one of the key components of comprehensive BC treatment. However, while chemotherapeutic agents effectively kill tumor cells, they also damage normal tissues and cells, leading to a range of adverse effects. A clinical study involving 26 BC patients demonstrated that higher gut microbiota diversity is associated with improved outcomes in neoadjuvant chemotherapy (Li et al., 2022). In patients who did not respond to neoadjuvant chemotherapy, the types of intestinal microbiota decreased. The regulation of the intestinal microbiota on host CD4^+^ T lymphocytes may affect the sensitivity and pathological physiological response to neoadjuvant chemotherapy. The β-diversity of the gut microbiota can predict the main adverse neurological reactions after chemotherapy, including sensory abnormalities, peripheral sensory neuropathy, memory impairment, and attention deficit (Terrisse et al., 2021). Microbial metabolites serve as key hubs linking the gut microbiota to cancer, regulating cancer progression by reshaping the tumor microenvironment, modulating immune cell function, and influencing cytokine levels. These metabolites regulate signaling pathways such as MAPK, PI3K/Akt, and Wnt, thereby affecting tumor cell proliferation, apoptosis, and metastasis, while reducing treatment-related side effects and enhancing the efficacy of radiotherapy and chemotherapy (Yang et al., 2023).

### 3.2 The interaction between gut microbiota and endocrine therapy

Endocrine therapy is a key treatment approach for hormone receptor-positive BC. Tamoxifen, one of the most commonly used endocrine therapy agents, is an anti-estrogen drug that acts as a competitive inhibitor of estradiol, thereby blocking its effects on target tissues. The gut microbiota plays a crucial role in tamoxifen metabolism, as it facilitates the conversion of tamoxifen into active metabolites—4-hydroxytamoxifen and endoxifen—enabling its anti-tumor activity (Arnone and Cook, 2022). Imbalance of the gut microbiota may lead to abnormal metabolism of tamoxifen, affecting the generation of its active metabolites, and thereby reducing the efficacy of endocrine therapy.

### 3.3 Potential role of gut microbiota in immunotherapy

Immunotherapy, as an emerging cancer treatment method, has brought new hope to BC patients. The gut microbiota plays a potentially significant role in immunotherapy by modulating the host immune system and influencing treatment efficacy. Specific gut bacteria can activate immune responses, enhance the capacity of immune cells to recognize and eliminate tumor cells, thereby improving immunotherapeutic outcomes. The composition of the gut microbiota affects the effectiveness of anti-cancer immune surveillance as well as the therapeutic responses to immune checkpoint inhibitors targeting the CTLA-4 or PD-1/PD-L1 pathways and to immunogenic chemotherapy (Routy et al., 2018). Among the patients whose conditions have responded well to treatment, the bacterial communities are highly diverse and contain specific bacterial species such as *Akkermansia muciniphila*, *Prevotella*, *Bifidobacterium* and *Peptococcus* (Routy et al., 2018). In mouse and patient-derived organoid models, IPA improved the responsiveness to anti-PD-1 therapy at the pan-cancer level, including melanoma, BC and colorectal cancer (Jia et al., 2024). Moreover, when fecal samples from patients who responded to immunotherapy were transplanted into mice, the efficacy of anti-tumor immunotherapy was enhanced, and the level of CD8^+^T cells increased (York, 2018). In contrast, the level of inhibitory CD4^+^T cells in FMT mice that received unresponsive patients rose (York, 2018). This indicates that the gut microbiota can regulate the anti-PD1 efficacy through interaction with the host immune system, and is expected to become a new therapeutic target. Therefore, future research should focus on integrating multi-omics approaches with well-designed animal models to elucidate the causal roles and mechanistic pathways of key functional microbes and their metabolites in modulating treatment responses. Concurrently, large-scale clinical cohort studies are needed to establish standardized protocols for integrating microbiota-targeted interventions with conventional therapies and to rigorously assess their long-term safety and clinical efficacy, thereby facilitating the translation of these strategies from bench to bedside.

## 4 Strategies for regulating gut microbiota to intervene in BC

The gut microbiota can exert diverse preventive and therapeutic effects on BC development. Targeted modulation of the gut microbiota represents a promising strategy for the prevention and treatment of BC. Dietary interventions, probiotics and prebiotics, and FMT are currently considered effective approaches, although further clinical studies are required for validation. In addition, the rational use of antibiotics, supplementation of probiotics, and modulation of the microbiota by traditional Chinese medicine extracts hold potential therapeutic value. Future efforts should focus on integrating existing therapies to enable individualized, multi-target interventions.

### 4.1 The influence of dietary regulation on gut microbiota and BC

**Figure 2:**
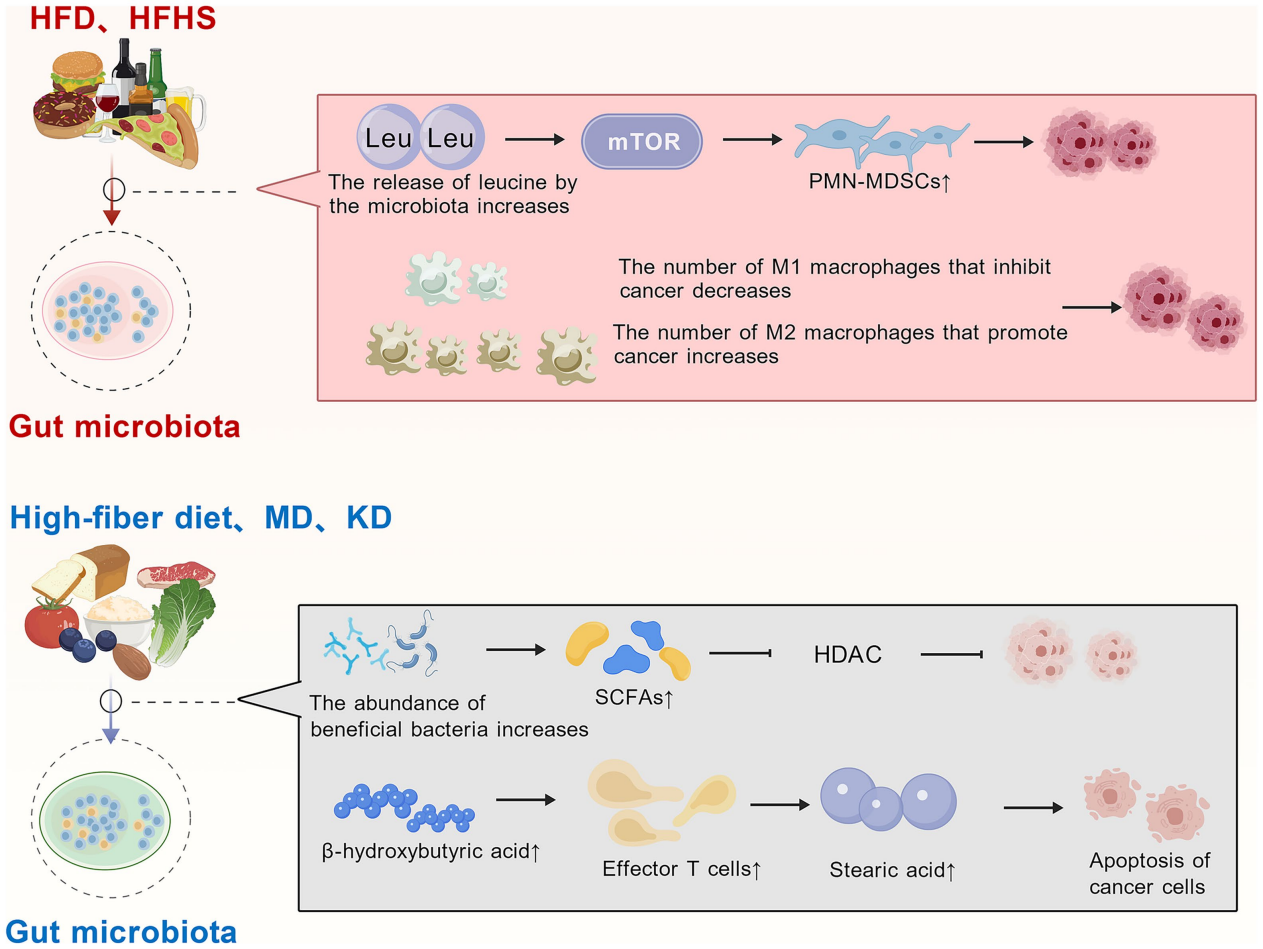
Dietary patterns influence the gut microbiota and thereby affect tumor development. HFD disrupts the microbial balance and promote the progression of tumors, such as BC. In contrast, interventions including intermittent fasting, high-fiber diets, the MD, and the KD can exert anti-tumor effects by modulating the gut microbiota, promoting the production of beneficial metabolites, or enhancing immune system activation (all figures are created with BioGDP.com).

Diet is associated with various diseases, and different dietary patterns can influence the host’s gut microbiota. In a retrospective study involving nearly 20,000 patients with early-stage BC, obesity at the time of diagnosis was found to increase the risk of distant metastasis by 46% within 10 years and the risk of BC–related death by 38% over a 30-year period (Ewertz et al., 2011). Long-term high-fat diet (HFD) disrupts the balance of the gut microbiota and accelerates tumor growth. An HFD alters the gut microbiota, leading to increased leucine release by gut microbes. This, in turn, activates the mTOR signaling pathway in myeloid progenitor cells, enhancing the production and differentiation of polymorphonuclear myeloid-derived suppressor cells (PMN-MDSCs), thereby promoting cancer progression (Chen et al., 2024) (Figure 2). Similar to HFD, high-fat high-sucrose diet (HFHS) reduces tumor invasion of M1 macrophages, promotes tumor invasion of M2 macrophages, inhibits inflammatory response and promotes tumor growth (Imbroisi Filho et al., 2021) (Figure 2). On the contrary, proper fasting can inhibit tumor cell proliferation. It also induces DNA damage and oxidative stress, activates the immune system—including T cells and natural killer (NK) cells—and modulates the composition of the gut microbiota to exert anti-tumor effects (Vernieri et al., 2024).

Therefore, modifying diet to restore gut microbiota may help curb tumor growth. Soluble fiber is significantly negatively associated with BC, high-fiber diet decreases the risk of BC (Farvid et al., 2020). Dietary fiber inhibits tumor through producing SCFAs, and adequate dietary fiber reduce the risk of death from diseases by 30% (Spencer et al., 2021; Abdeen et al., 2025). Mediterranean diet (MD) is rich in whole grains, fish, nuts, fruits and vegetables, which improve obesity-related factors including gut microbiota disorders (Almanza-Aguilera et al., 2023; Bolte et al., 2023). Adherence the MD can enhance anti-tumor immune responses and reduce the risk of several cancer types, including BC, lymphoma, colorectal cancer, and esophageal cancer (Castro-Espin et al., 2023; Solans et al., 2019; Jones et al., 2017; Schulpen et al., 2019). Besides, ketogenic diet (KD) restrains the progress of cancer in mice through T cells-dependent manner (Ferrere et al., 2021) (Figure 2). Recent findings indicate that the gut microbiota in mice on a ketogenic diet, which produces stearic acid, significantly increases and clearly suppresses tumor growth (Tsenkova et al., 2025) (Figure 2). Stearic acid directly induces apoptosis of cancer cells, and it also decreases the cancer-promoting Th17 immune cells (Tsenkova et al., 2025). In conclusion, various dietary patterns influence the composition of the gut microbiota, which in turn affects tumor development.

### 4.2 The application prospects of probiotics and prebiotics

The advantages of probiotics over traditional BC treatments are that they can better target cancer cells and have fewer side effects. Some probiotics, such as *Bifidobacterium*, *Lactobacillus*, *Propionibacterium*, and *Streptococcus thermophilus*, are effective in improving the prognosis of cancer patients when combined with monoclonal antibodies (PD-1/PD-L1, CTLA4) (Li et al., 2022). Probiotics (*Lactobacillus brevis* and *Lactobacillus casei*) have been found to inhibit breast tumor cells growth and reduce tumor volume through their immunomodulatory effects (Dameshghian et al., 2024). Moreover, probiotic supplementation helps maintain microbial diversity, the abundance of butyrate-producing bacteria and *Bifidobacterium*, and further alleviates diarrhea caused by BC chemotherapy. In addition, metabolites of the gut microbiota, such as IPA, exhibit significant anti-tumor effects. IPA inhibits the proliferation of murine 4 T1 cells, increases cellular oxidative and energetic stress, and reduces the number of cancer stem cells (Sári et al., 2020). Common prebiotics include inulin and fructooligosaccharide, they promote the growth of probiotics and metabolites to act the effect of anti-tumor (Li et al., 2022). Researchers have confirmed that nanoparticles loaded with capecitabine were constructed using the prebiotic xylan-stearate conjugate to promote the treatment of colorectal cancer (Lang et al., 2023). Prebiotics mainly produce SCFAs, including acetate, propionate, butyrate.

### 4.3 Research progress of fecal microbiota transplantation in the treatment of BC

The FMT is a biological treatment technology that involves transplanting functional microbiota from the feces of healthy donors into the patient’s intestinal tract to restore the balance of the intestinal microbiota and thereby treat diseases associated with microbial dysbiosis. FMT regulates intestinal homeostasis and immune balance by improving the composition of gut microbiota, thereby changing TME, enhancing the effect ICI (Yang et al., 2024). FMT has shown good effect in the study of digestive tract tumors and a variety of non-digestive tract tumors (Yang et al., 2024). FMT changes cancer patients gut microbiota, and enhances the function Of PD-1 (Biancheri et al., 2018). The researchers transplanted the gut microbiota of patients who responded to immunotherapy into those who did not, the results indicate that some subjects returned to responding to immunotherapy (Biancheri et al., 2018). Thirteen patients with advanced solid tumors who were resistant to PD-1 blockade received FMT from responders and were subsequently rechallenged with anti-PD-1 therapy. Six patients exhibited microbial alterations and clinical benefits. The objective response rate and disease control rate were 7.7 and 46.2%, respectively. This clinical study suggests that the combination of FMT and anti-PD-1 therapy may help overcome resistance to anti-PD-1 treatment in patients with advanced solid tumors (Kim et al., 2024). A similar conclusion was also reached in another clinical trial on melanoma (Hadi et al., 2025). These evidences suggest that FMT is a new target for cancer treatment and holds promising application prospects for the prevention and treatment of BC. However, additional clinical trials are still required to establish the indications and safety profile of FMT in patients with hematological malignancies.

### 4.4 Other treatment methods for regulating the gut microbiota

For BC patients who require antibiotic use due to their clinical condition, appropriate probiotic supplementation can be administered concurrently with or after antibiotics to mitigate antibiotic-induced damage to the gut microbiota. Nebulized treatment with vancomycin or neomycin reduces bacterial load in the lungs of mice, decreases regulatory T cell activity, enhances effector T cell and NK cell activity, and inhibits melanoma lung metastasis (Le Noci et al., 2018). The anti-tumor efficacy of antibiotics primarily depends on the reduction of IL-17A, this depletion results in an increase in anti-tumor T cells secreting IFN-*γ* within the tumor microenvironment and a decrease in pro-tumor T cells producing IL-17A and IL-10 (Sethi et al., 2018).

Some components of traditional Chinese medicine can promote the growth of beneficial intestinal bacteria, inhibit the proliferation of harmful bacteria, and modulate the metabolic functions of the gut microbiota, thereby exerting positive effects on BC treatment. Pien Tze Huang, a traditional Chinese medicine, increases the levels of beneficial metabolites such as taurine and hypotaurine, bile acids, and unsaturated fatty acids, and significantly restores intestinal barrier function. It inhibits the PI3K-Akt, IL-17, TNF, and chemokine signaling pathways, thereby suppressing colorectal tumorigenesis (Gou et al., 2023). Using lentinan polysaccharide (LNT) and ursolic acid (UA), self-assembled nanomedicine LNT-UA was prepared. LNT-UA more effectively induced cell apoptosis and activated immunogenic cell death in colorectal cancer cells, and showed significant anti-tumor effects in CRC mouse models (Mao et al., 2022).

Dietary modifications, prebiotics, probiotics, FMT, and traditional Chinese medicine—among other interventions targeting the gut microbiota—hold promise for the prevention and treatment of breast cancer. Next, multi-omics technologies can be employed to elucidate the molecular mechanisms underlying these interventions, with a focus on identifying optimal patient populations for specific therapies such as probiotics and FMT, and on establishing a robust biomarker system. Ultimately, this may pave the way toward truly precise and effective individualized microbiota-targeted therapies.

## 5 New insights into targeting gut microbiota for the prevention and treatment of BC

The traditional classification of BC primarily relies on the expression status of hormone receptors (ER, PR) and HER2. However, considerable variability in treatment response and prognosis persists among patients assigned to the same molecular subtype. The gut microbiota may serve as a critical “hidden variable” contributing to these interindividual differences. By integrating microbiota-related characteristics, we aim to develop a precise classification system based on gut microbiota features, which could enable more accurate and targeted therapies for BC.

**Figure 3:**
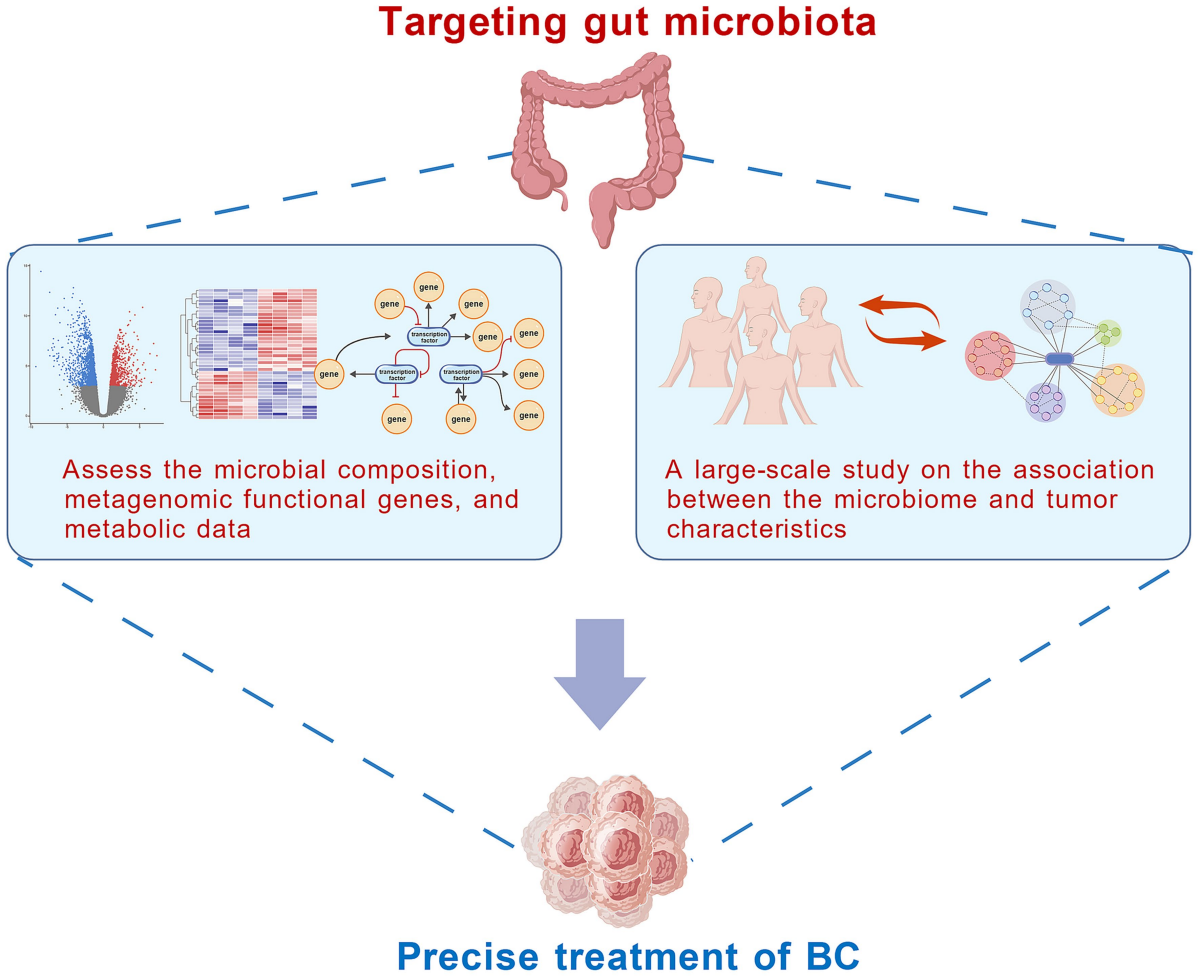
New insights into targeting gut microbiota for the prevention and treatment of BC. By integrating the composition of gut microbiota, metagenomic functional genes and metabolomics data to construct a three-dimensional characteristic spectrum of “microbiota – genes – metabolites,” and combining with a large sample cohort to clarify the association between microbiota subtypes and tumor characteristics, a precise BC typing system based on gut microbiota characteristics can be established to improve the limitations of traditional typing (all figures are created with BioGDP.com).

This approach requires not only an assessment of microbial composition, but also an integration of metagenomic functional genes (e.g., β-glucuronidase encoding gene, SCFA synthesis genes) and metabolomics data (e.g., levels of estradiol metabolites, SCFAs, and secondary bile acids), to construct a comprehensive “microbiota–gene–metabolite” three-dimensional profile (Figure 3).

Furthermore, large-scale cohort studies can help clarify the associations between distinct microbiota subtypes and tumor characteristics, such as invasiveness, metastatic potential, and risk of treatment resistance (Figure 3). For instance, a microbiota subtype characterized by a high abundance of pro-inflammatory bacteria (e.g., *E. coli*) and low levels of anti-inflammatory species (e.g., *Faecalibacterium*) may correspond to an immunosuppressive tumor microenvironment and a phenotype resistant to chemotherapy. Conversely, a microbiota subtype enriched in butyrate-producing bacteria may be associated with enhanced tumor immunogenicity.

## 6 Conclusion

The BC poses a serious threat to women’s health, with advanced-stage disease associated with high mortality due to widespread metastasis. In recent years, the gut microbiome has garnered considerable attention as a key contributor to the progression of various diseases. Accumulating evidence suggests that the gut microbiota may influence BC development through multiple mechanisms, including estrogen metabolism, dietary patterns, and immune system modulation. Probiotic and prebiotic supplementation show potential in inhibiting tumor progression, and dietary improvements may contribute to breast cancer prevention. Nevertheless, comprehensive data elucidating the relationship between the gut microbiome and breast cancer remain limited. Tailoring treatment strategies based on gut microbiota profiles may improve patient prognosis. A deeper understanding of the complex interactions between the microbiome and tumor biology could not only clarify the underlying mechanisms of BC but also provide novel insights into more precise and effective preventive and therapeutic approaches.

### 6.1 Limitations and future directions

Although the role of the gut microbiota in the development and treatment of BC has been extensively documented, causal evidence linking gut microbiota dysbiosis to BC remains lacking. Furthermore, substantial inter-individual variations in microbial composition, along with confounding factors such as diet and geographic location, may limit the generalizability of research findings. Consequently, current evidence primarily provides hypotheses and potential directions for future investigation, falling short of direct application in clinical diagnosis and therapy. Future studies should integrate large-scale clinical cohort analyses with well-designed animal models to establish causal relationships and comprehensively investigate the specific mechanisms by which the microbiota influences BC through the “gut-mammary” axis. Particular emphasis should be placed on elucidating the mechanistic roles of microbial metabolites—such as SCFAs and secondary bile acids—in modulating both the local tumor immune microenvironment and systemic estrogen levels.
